# Smartphone-Based Estimation of Item 3.8 of the MDS-UPDRS-III for Assessing Leg Agility in People With Parkinson's Disease

**DOI:** 10.1109/OJEMB.2020.2993463

**Published:** 2020-05-08

**Authors:** Luigi Borzì, Marilena Varrecchia, Stefano Sibille, Gabriella Olmo, Carlo Alberto Artusi, Margherita Fabbri, Mario Giorgio Rizzone, Alberto Romagnolo, Maurizio Zibetti, Leonardo Lopiano

**Affiliations:** Department of Control and Computing EngineeringPolitecnico di Torino 19032 10138 Torino Italy; Department of Neuroscience “Rita Levi Montalcini,”University of Turin 9314 10124 Torino Italy

**Keywords:** Artificial neural networks, bradykinesia, leg agility, parkinson's disease, smartphone

## Abstract

*Goal:* In this paper we investigated the use of smartphone sensors and Artificial Intelligence techniques for the automatic quantification of the MDS-UPDRS-Part III Leg Agility (LA) task, representative of lower limb bradykinesia. *Methods:* We collected inertial data from 93 PD subjects. Four expert neurologists provided clinical evaluations. We employed a novel Artificial Neural Network approach in order to get a continuous output, going beyond the MDS-UPDRS score discretization. *Results:* We found a Pearson correlation of 0.92 between algorithm output and average clinical score, compared to an inter-rater agreement index of 0.88. Furthermore, the classification error was less than 0.5 scale point in about 80% cases. *Conclusions:* We proposed an objective and reliable tool for the automatic quantification of the MDS-UPDRS Leg Agility task. In perspective, this tool is part of a larger monitoring program to be carried out during activities of daily living, and managed by the patients themselves.

## I. Introduction

Parkinson’s disease (PD) is one of the most common neurodegenerative disorders [1], with 7–10 million affected people worldwide and a prevalence exceeding 1.9% over the age of 80 [2]. It is characterized by both motor and non-motor signs and symptoms, related to the degeneration of dopamine neurons, particularly in the area of the brainstem called *substantia nigra pars compacta*. After more than forty years since its introduction in the clinical practice, Levodopa (L-dopa) is still the gold standard for the control of PD motor symptoms [3]. Yet, L-dopa has introduced an additional source of features into the natural evolution of PD through its potential to induce involuntary movements (i.e. dyskinesias) and motor response fluctuations. A timely, objective monitoring of motor fluctuations can represent a precious piece of information for the clinicians, because it enables a drug posology adaptation to the specific response of each single patient. However, motor fluctuations are difficult to appreciate in a medical office. Outpatient visits are scheduled once a year and have limited duration, hence only gross variations are appreciated. Moreover, the visit itself may affect the actual patient status, which is conditioned by the time interval elapsed since the last drug administration, the general health conditions and many other subtle factors [4]. This makes it hardly possible for the neurologist to appreciate short-term variations in order to plan fine adjustments of the pharmacological treatment. Our work finds its ultimate motivation in the necessity of an *electronic diary* for quantitative assessment of the motor conditions of PD patients. We believe that this tool, in order to achieve large-scale application, should make use of cheap, easy-to-use and widespread instrumentation, such as smartphones. It could enable a better follow-up and provide effective and supportive treatment, accessible to all patients also in a context of overall cost reduction. This fits the Digital Health Pathways [4], a pipeline defining guidelines for a patient-centered platform exploiting wearable devices to monitor disease progression not only in controlled clinical environment. At present, the MDS-UPDRS (Movement Disorder Society – Unified Parkinson's Disease Rating Scale) [5], [6] is universally employed to assess the course of PD after diagnosis. The evaluation encompasses six parts. Part III, which is the most relevant for this work, is the clinical evaluation of several motor skills. The objective of our work is to face the following questions. Is it possible to automatically evaluate (at least a subset of) MDS-UPDRS-part III items using inertial data gathered from the sensors embedded in a common smartphone? What is the achievable precision of this estimation? As a starting point, in this paper we focus on Leg Agility (LA), a task included in the MDS-UPDRS scale for motor evaluation of lower limbs (task 3.8). LA consists of raising and stomping the foot on the ground at least 10 times, as high and fast as possible, starting from a sitting posture. Each leg is tested separately.We have selected this item due to its simple and safe execution and relatively easy assessment. Nevertheless, LA is correlated to bradykinesia and to the fluctuating patient's response to drugs [7]. Hence, the automatic estimation of LA task yields intrinsically useful clinical information. We use a dedicated smartphone to collect accelerometer and gyroscope data during the execution of LA, and evaluate several Machine Learning (ML) techniques with respect to the capability of replicating the clinical MDS-UPDRS score. In this preliminary stage, we have gathered data in controlled conditions, i.e. during pre-scheduled outpatient visits. However, the achieved results allow us to proceed with the evaluation in non supervised environments. Considerations on MDS-UPDRS scoring could be find in Section V-A. Several papers address data mining and Artificial Intelligence (AI) techniques to recognize the severity of PD cardinal motor signs, using data derived from wearable sensors. In Section V-C, main information about dataset, methods and results of most relevant works are briefly described. All the considered studies present some limitations: reduced sample dimension (maximum 44 PD subjects), dedicated hardware (only one study employs smartphones), number of sensors (most studies use three sensors). Furthermore, some relevant dataset details (e.g. cardinality of MDS-UPDRS classes) are not reported. The rest of this paper is organized as follows. In Section II we describe the experimental setup and the cohort of people with PD enrolled for this experiment, as well as the ML algorithms implemented. In Sect III the achieved results are described and discussed, and in Section IV conclusions are drawn.

## II. Materials and Methods

**Fig. 1.**
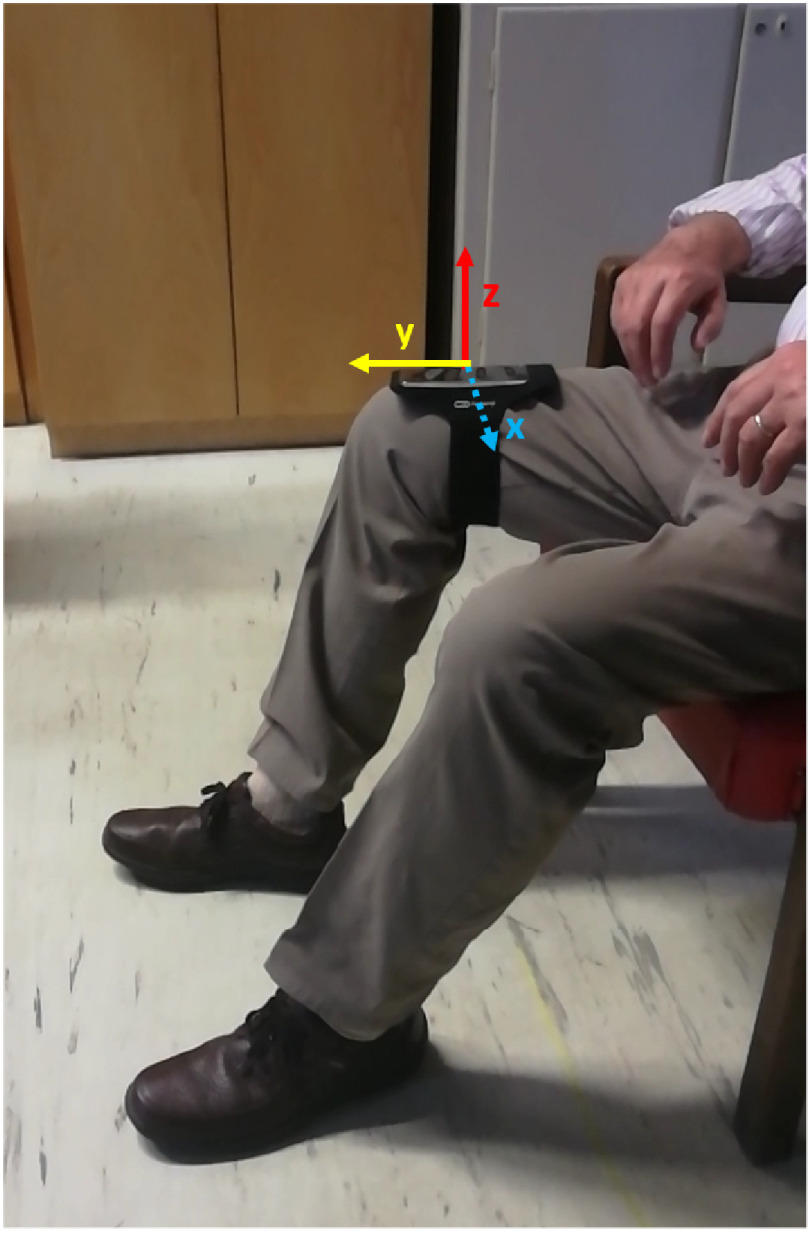
Smartphone position adopted for the LA task scoring.

The experiments have been carried out at the University Hospital *Città della Salute e della Scienza*, Turin (Italy), which hosts the Regional Reference Center for Parkinson's Disease and Movement Disorders. The study has been conducted in accordance with the Declaration of Helsinki and approved by the local Ethics Committee (Ethics approval number 1534-2019). Participants received detailed information on the study purposes and execution, and written informed consent for observational study was obtained. Demographic and clinical data were noted anonymously. Patients agreed to the video-taping of the procedure after receiving suitable explanations and being guaranteed that he/she cannot be identified (only the patient's legs were video-captured, as in Fig. 1) and the videotapes are not made available to persons different of the authorized ones. The experiments were carried out in hospital during the periodically scheduled outpatient visits; hence, the patients’ safety was guaranteed by the presence of the medical staff.

### A. Data Collection

A total number of 93 people with PD were recruited in the study. The inclusion criteria were: a clinical diagnosis of Parkinson's disease with motor signs and symptoms [8], no major cognitive impairment or other conditions preventing the patient from correctly accomplishing the task. Given that data acquisition was performed during pre-scheduled outpatient visits, most patients were in *daily on* condition, i.e. they had taken their usual drug dose, even though different time intervals had elapsed since then, and the next scheduled dose was not imminent. In few, particular cases (visit scheduled late in the morning – about 4%), some of them showed some end-of-dose effect. Yet, the number of these patients was not enough to perform differential analysis, thus we chose not to differentiate patients based on motor condition. We believe that this does not affect in either sense the system performance, due to the small number of subjects. The population characteristics are summarized in Table I.

**TABLE I:** Demographic and Clinical Characteristics of PD Population

**# patients**	**Age** **(mean $\pm$ SD)**	**Years** **from diagnosis** **(mean $\pm$ SD)**	**Hoehn and Yahr** **(mean $\pm$ SD)**	**LA score** **(mean $\pm$ SD)**
93 (70% male)	69 $\pm$ 10	9.0 $\pm$ 6.5	2.5 $\pm$ 0.8	2 $\pm$ 1

During their visit, the subjects were asked to sit in a straight-backed chair and place the foot on the ground in a comfortable position. Then, after being properly instructed by an expert neurologist as recommended in the MDS-UPDRS guidelines, they performed LA with each leg separately. A simple Velcro armband equipped with the smartphone was placed around the patient's thigh, with the y-axis parallel to the femur direction. The smartphone recording application was started before and stopped after the execution of the task, thus each recording included a signle LA execution. A preliminar analysis on LA data from young control subjects is reported in Section V-B, meant to verify the suitability of smartphone for the specific data acquisition task. Experiments were video recorded in order to allow multi-rater evaluation. Fig. 1 shows the experimental setup. Globally, we have measured 184 LA test (2 patients were able to perform the test with a single leg only).

**Fig. 2.**
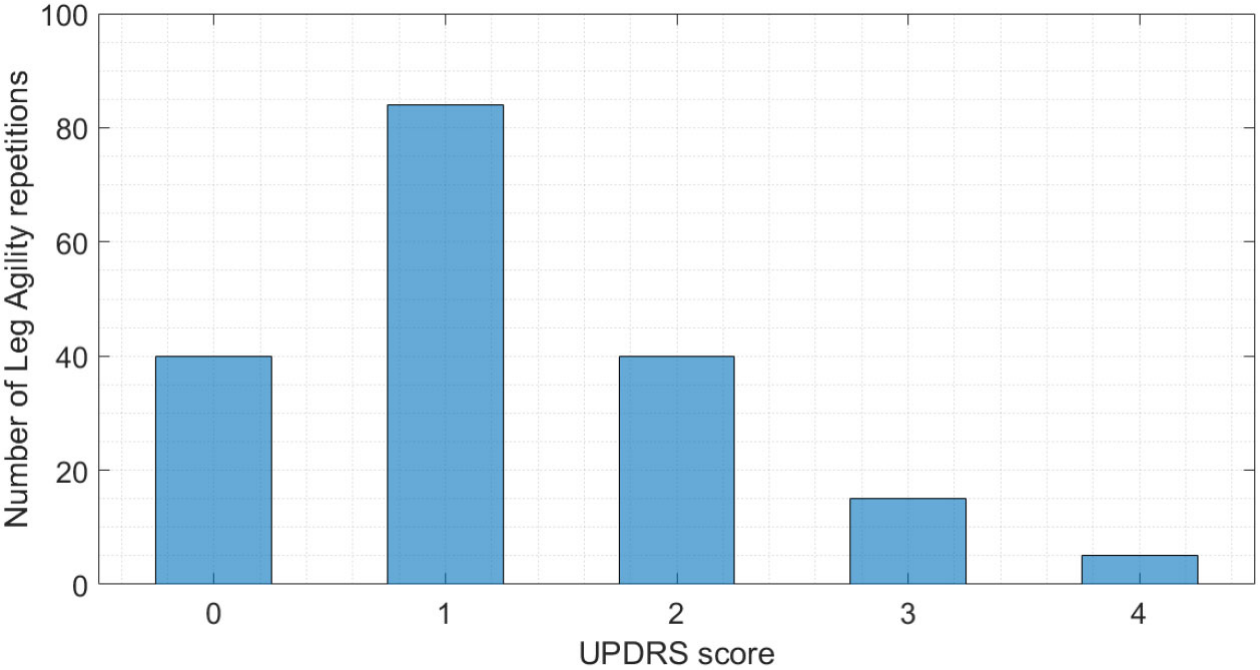
Distribution of the MDS-UPDRS scores assigned to the LA tasks. 0: normal. 1: slight. 2: mild. 3: moderate. 4: severe.

The LA task was scored by four expert neurologists according to the MDS-UPDRS scale, either directly or after inspection of the video sequences. The rounded average ratings have been employed as the class labels for the supervised classification algorithms. Fig. 2 shows the distribution of the assigned MDS-UPDRS scores. It is worth noticing that the dataset encompasses few cases in classes MDS-UPDRS-3 and 4. Actually, the clinical conditions of patients belonging to such classes are severe, and may even prevent them from executing the task. In particular, even though we have been actually able to test five MDS-UPDRS-4 patients, the usefulness of including them in a monitoring system is questionable. Therefore, as also suggested by the expert clinicians, we excluded MDS-UPDRS-4 patients from the subsequent analysis, and reported exclusively for visualization. Acceleration, angular velocity and orientation data have been collected by means of SensorLog App, stored locally on a SD card, exported in CSV format and processed offline using MATLAB, version 2018a for Windows 10.

### B. Feature Extraction and Selection

Once the data have been registered, signals have been recalibrated in order to compensate for deviations from the ideal positioning (i.e. gravity acting only on the vertical component, i.e. the z-axis of the accelerometer). The method proposed in [9] was applied, consisting of 3-axis accelerometer orientation correction by applying a quaternion rotation transformation to the device raw data. After mean value removal, pitch, acceleration and angular velocity have been lowpass filtered in order to remove high frequency noise. A 2-order zero-lag low-pass Butterworth filter with a cutoff frequency of 4 Hz was chosen, in order to keep at least 90% of the signal power (computed using the Fast Fourier Transform – FFT – on all data). Then, a set of 36 kinematic features (reported in Table II) have been extracted from each signal, representative of the major traits that distinguish motion in people with PD and unaffected controls. Insights provided by the literature on other similar studies [10]–[14] have been taken into account.Besides cross-correlation, which provides information in the spectral domain, we also computed signal Fast Fourier Transform (FFT), and we extracted features as frequency, amplitude and width of the dominant harmonic, total number of harmonics, power ratio between principal and other harmonics. Then we further combined some features, in order to increase their discriminating power (e.g. ‘Mean peak value’ feature takes in consideration the number and the amplitude of harmonics in the FFT).

**TABLE II:** List of Features Extracted in this Work, Along With the Selected Components

**Selected component**	**Extracted features**
$\theta _{x}, \omega _{x}, \alpha _{z}$	Dominant frequency
/	Entropy
/	Minimum
$\omega _{x},\alpha _{z}$	Maximum
$\omega _{x}, \alpha _{z}$	Root Mean Square
$\omega _{x},\alpha _{z}$	Range
$\omega _{x},\alpha _{z}$	Spectral Entropy
$\omega _{x},\alpha _{z}$	Mean amplitude
$\omega _{x},\alpha _{z}$	Regularity
$\alpha _{z}$	Dominant Ratio
/	Standard deviation
/	Mean peak value

$\theta _{x}$: Pitch Signal Around x-axis. $\omega _{x}$: Angular Velocity Around x-axis. $\alpha _{z}$: Acceleration Along Vertical Direction. / Indicates That None of the Components Have Been Selected

**Fig. 3.**
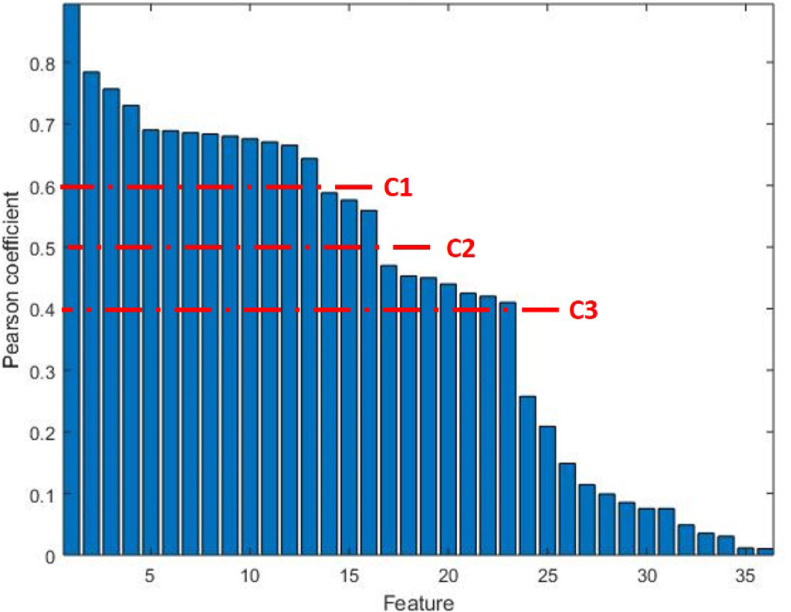
Feature ranking based on Pearson's correlation coefficient (r). *C1, C2, C3* identify gaps in r-values of adjacent features.

In order to identify the most meaningful features, we performed a feature selection based on the correlation between feature values and target. Fig. 3 reports the Pearson's correlation coefficient for each feature.

The optimal features subset, i.e. that containing the most informative features while mantaining a reduced dimension, is obtained as follows. We first sorted features in descending order of Pearson correlation value, and features exhibiting a correlation lower than 0.4 (i.e. weak correlation) were discarded. Then, we set three thresholds, referred in Fig. 3 as *C1, C2* and *C3*, corresponding to gaps in correlation value between adjacent features. The three resulting feature subgroups were given as input to common ML algorithms (listed in Section II-C). The final subset was that leading the higher accuracy; *C2* turned out to be the optimal value, yielding to 16 selected features (see Table II). We also checked that the correlation between the selected features was not higher than the correlation with the target, in order to not include redundant features in the final set. A brief description of some features is provided below.

**Dominant frequency.** It is the frequency value corresponding to the highest peak of the Fast Fourier Transform (FFT) function.

**Spectral Entropy.** It is the Shannon entropy computed on the FFT of the signal.

**Regularity.** It is the amplitude of the first positive peak of the autocorrelation function, normalized to the maximum of correlation function, as described in [15].

**Dominant Ratio.** It is the ratio of the dominant frequency band power to total power.

### C. Addressed ML Algorithms

Due to the many factors affecting the performance, actually no ML algorithm outperforms the others for every problem at hand [16]. Hence, a sound approach is to test several ML methods and select the best one for the specific problem to be solved. We employed common ML algorithms to compare with other studies (e.g. [14], [17], [18]). Moreover, we decided to also implement a novel Artificial Neural Network approach, yielding a continuous output. To identify the best combination of each ML model parameters, we employed a bayesian optimization algorithm. We set the cross-validation error as objective function to minimize in a Leave-One-Subject-Out (LOSO) validation. As for the hyperparameters to optimize, we selected main parameters from all eligible in each model, namely: kernel function, kernel scale and boxconstraint for one-vs-one SVM; number of neighbors, distance metric and distance weight for KNN; maximum number of splits and split criterion for DT. As for ANN, we set stop conditions to max 2000 iterations and gradient value to $10^{-5}$, starting learning rate to 0.01, increasing and decreasing values to 10% and 20%, respectively. Then, we tuned the number of hidden layers, number of hidden neurons per layer and transfer function. Finally, we selected the ANN architecture returning the lowest misclassification error (i.e. that providing the best accuracy). The final parameter selection for each addressed algorithm turned out to be as described below.

• **SVM.** Kernel function: linear, boxconstraint: 36.
• **kNN.** Number of neighbors:5, distance metric: euclidean, distance weight: equal.
• **DT**. Split criterion: Gini-Simpson diversity index [19], maximum number of split equal: 4.
• **ANN.** Number of hidden layers: 2, number of neurons per layer: 16, transfer function: hyperbolic tangent sigmoid.

## III. Results and Discussion

In this section, we present the classification results achieved by the various ML algorithms addressed in Section ?? for MDS-UPDRS LA score estimation. Furthermore, we focus on the inter-rater variability issue, and propose a possible solution.

### A. Classification Results

The feature set, identified as discussed in Section II, is input to each classification algorithm. A LOSO validation criterion has been employed, i.e. each element of the data set is used to test the performance of each algorithm, trained using the remaining elements.

Table III summarizes the performance of each model in terms of accuracy and Area Under the Curve (AUC).

**TABLE III:** Performance of Several ML Methods in Case of Discrete Output

**Method**	**Accuracy (%)**	**AUC**
DT	59.1	0.53
kNN	60.3	0.82
SVM	60.9	0.80
ANN	77.7	0.92

It can be appreciated that the ANN model exhibits the best performance among the implemented ML classifiers (accuracy 77.7%), outperforming the results reported in literature (i.e. in [14], [17] an accuracy of 43% is reported). In order to get further insight in the behaviour of the proposed classifiers, we computed the Cumulative Distribution Function (CDF) as a function of the absolute error, i.e. the absolute difference between the MDS-UPDRS class yielded by the algorithm and the rounded average MDS-UPDRS class provided by the neurologists. All methods, except DT, classify incorrectly only by maximum one step on the MDS-UPDRS scale, i.e. classification error is $\leq 1$ in 100% of cases. Such an error is comparable with the inter-rater variability, as also discussed in [14]. In fact, the MDS-UPDRS evaluation performed by several neurologists is often non homogeneous, especially due to the difficulty in discriminating between adjacent classes in cases of intermediate gravity. In this study, the inter-rater agreement index turned out to range in $[0.74-0.88]$.

**Fig. 4.**
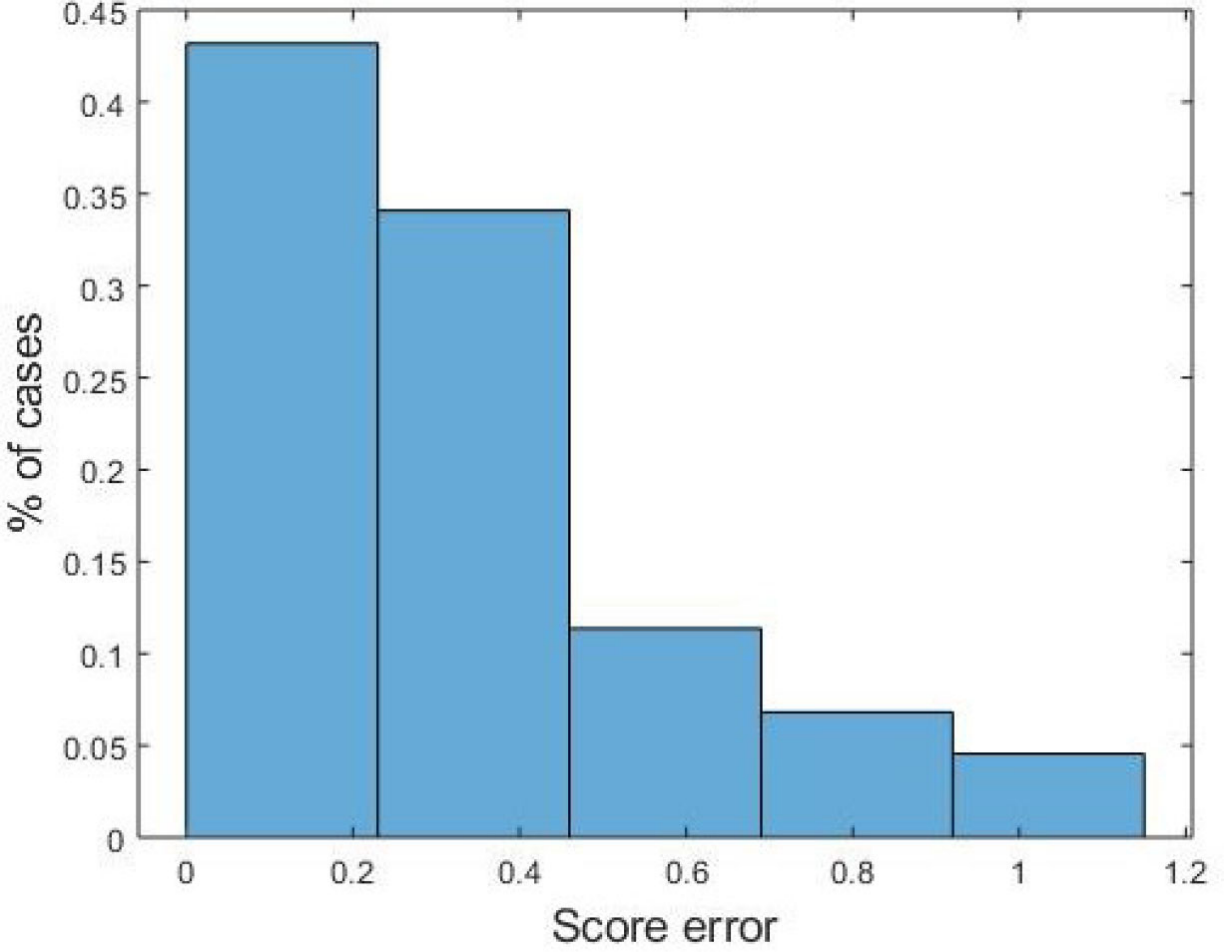
Histogram of distance between mean clinicians score and ANN outcomes. Continuous values were taken into account for assessment.

**Fig. 5.**
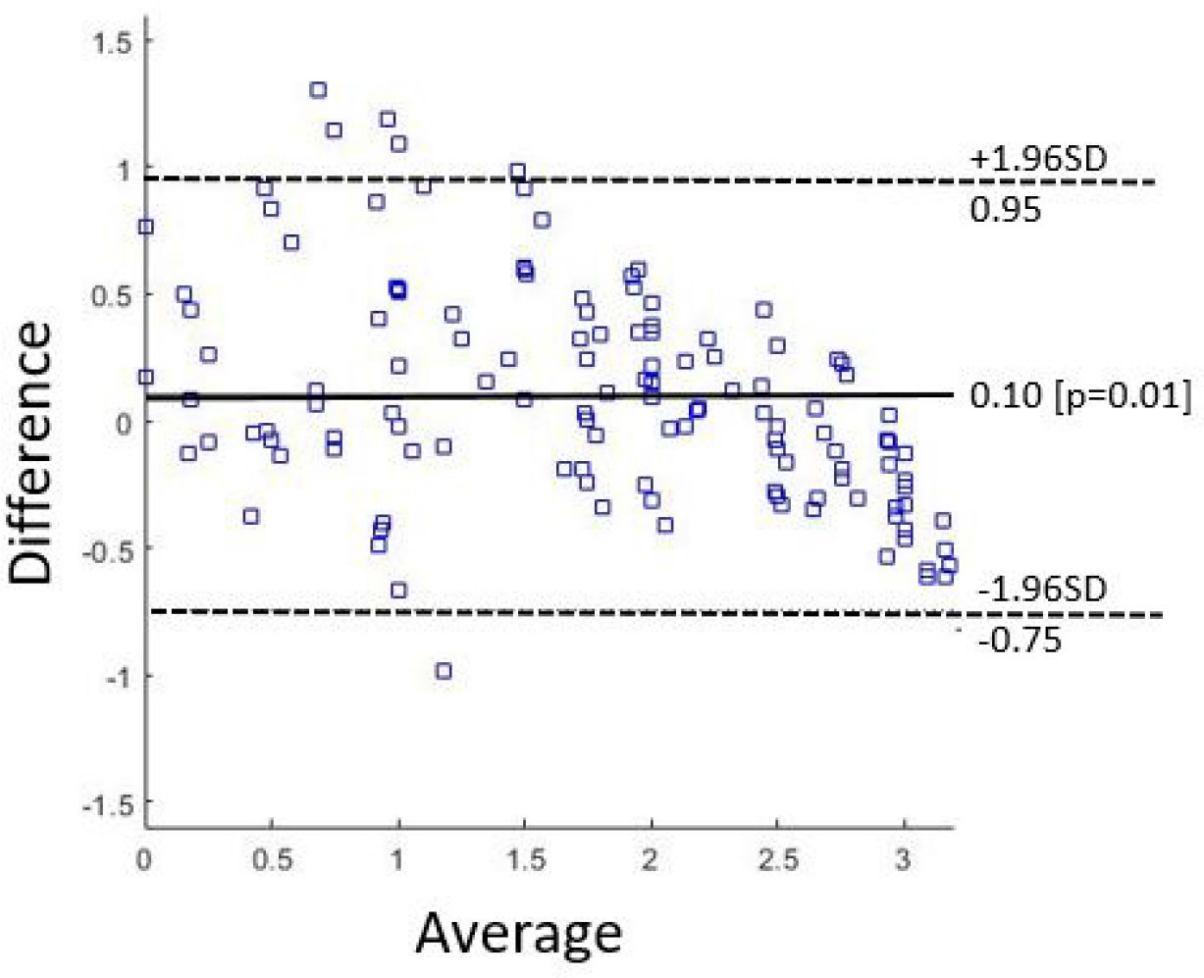
Bland-Altman Plot of mean clinicians score and ANN outcomes.

The ANN behaviour can be considered very good, significantly outperforming SVM and kNN. As for discrete scoring classification, the ANN error is $\leq 1$ in 100% of cases. Hence, ANN seems a good candidate to mimic the MDS-UPDRS clinical evaluation as for LA, with comparable reliability. As for the continuos output, the error histogram shown in Figure 4 reports an error $\leq 0.5$ in more than 80% of the istances. This makes us to claim than a finer discretization (i.e. 0.5 instead of unit steps) could certainly improve the algortihm performance. For the sake of completeness, the Bland-ALtman Plot reported in Figure 5 show the difference between outcomes of the implemented ANN model compared to the average of the four clinicians scores. Finally, in Table IV we compare our results with similar studies published in literature. As accuracy is a not sufficient measure of classification performance when dealing with unbalanced classes, we report other classification metrics previously introduced, namely Pearson's correlation coefficient $r$, Root Mean Square Error $RMSE$, Intra-Class correlation $ICC$. Moreover, these latter allow for a comprehensive comparison with other works.

**TABLE IV:** Comparison With State-of-the-Art Algorithms

**Study**	**Year**	**Device**	**#PD**	**#sensors**	**#raters**	***r***	**RMSE**	**ICC**
[20]	2012	IMU	42	5	3	0.79	0.46	–
[14]	2015	IMU	34	3	3	0.79	–	–
[21]	2015	Smartphone	14	1	$0^{\ast }$	0.5	–	–
[22]	2018	Smartphone	44	1	$0^{\ast }$	–	–	0.88
[18]	2019	IMU	19	2	3	0.83	0.53	0.89
[23]	2019	IMU	50	2	2	n.r. $^{\ast \ast }$	n.r. $^{\ast \ast }$	n.r. $^{\ast \ast }$
Proposed	2019	Smartphone	93	1	4	0.92	0.42	0.88

$^\ast$Dedicated Application-Performed Evaluation. $^{\ast \ast }$ Values not Explicitly Reported Within the Text.

From Table IV it can be noticed that the present work employs the largest patient cohort, as well as the higher number of clinicians; this ensures greater meaningfulness to the results. Furthermore, two out three performance metrics (i.e. $r$ and $RMSE$) outperform literature studies, while the $ICC$ coefficient is in line with the other studies. Specifically, higher Pearson's coefficient guarantees greater correlation with mean clinical score and a lower $RMSE$ is suggestive of a better concentration of data around the line of best fit. Finally, it is worth underlining that the correlation between our output and the mean clinical score is higher than the best clinicians agreement (i.e. 0.92 vs 0.88). The issue of inter-rater variability is discussed in the following.

### B. Discussion

The MDS – Task Force on Technology has recently published a document containing guidelines related to the correct use of technology for PD patient's monitoring and follow-up [24]. They strongly affirm that the actual state-of-the-art clinical assessment of this pathology, based on pre-scheduled medical examinations, leaves significant room for improvement. In fact, besides the sporadicity of outpatient visits, clinical scales, which are presently considered as the gold standard for PD monitoring, are prone to inter- and intra-rater variability, and their accuracy may be even outperformed by ML methods. Actually, clinicians could greatly benefit from reliable longitudinal data, collected in unsupervised environment during ADL, despite such data may be affected by several confounding variables. In order to make technology effective for patients, clinicians and caregivers, they propose a roadmap whose main objectives are diagnostic support, better patient's follow-up, and detection of subtle yet significant signs and symptoms of disease progression. An extended discussion on inter-rater variability and on the benefits that an objective tool could bring both to clinicians and patients, can be find in Section V-D. At present, data acquisition was performed during pre-scheduled outpatient visits. Most patients had taken their usual drug dose, even though different time intervals had elapsed since then, and the next scheduled dose was not imminent. In few, particular cases (visit scheduled late in the morning – about 4%), some of them showed some end-of-dose effect. Yet, the number of these patients was not enough to perform differential analysis, thus we chose not to differentiate patients based on motor condition. A possible alternative could be not to consider these few patients for analysis. Again, we believe that this does not affect in either sense the system performance, due to the small number of subjects.

## IV. Conclusion

In this paper, we have investigated the application of smartphone sensors for the evaluation of the LA task on people with PD. We have employed several ML algorithms for the task classification. At present, 93 patients have been evaluated by four neurologists during their scheduled outpatient visit, and their clinical assessment used to train the algorithms, as well as to benchmark their performance. The achieved results, in particular those yielded by ANN, are very promising, and make possible to devise a tool capable of monitoring fluctuations in the patient's motor conditions. We believe that a key feature of the proposed method is the fact that a common smartphone is employed. Due to its simplicity of use, large availability, low cost, this system can be self-managed by patients in his/her domestic environment, as also verified in a preliminary test. Moreover, exploiting a continuous score, physicians could monitor slight patient's fluctuations in more realistic conditions. Even though, at present, the processing is performed offline on a laptop, the low computational burden allow an easy integration in the same portable instrument.

Other future developments concern the acquisition of more training data, especially for the patients with severe conditions (MDS-UPDRS-3) and in non supervised conditions. Moreover, acquiring new data from a larger cohort of neurologists would allow an even stronger validation of the present results, enabling the use of the proposed tool in a clinical environment. Finally, we believe that phenotyping grouping (e.g. Tremor Dominant, PIGD, ID) would lead to interesting results. At present, we have not collected this kind of information, which goes beyond the goals of the study. Nevertheless, we plan to collect much more detailed clinical information in future works. Furthermore, we plan to perform data acquisition in both ON and OFF medication and to compare the results in different patients subgroups (e.g. based on drug posology or treatment type). As for an extreme practical use, we plan to test our tool in an experimental trial addressing the new drug opicapone [25], in order to measure to what extent this drug is able to limit daily fluctuations in people with PD.

At last, we also believe that, even though these results only address the LA, similar tests based on specific MDS-UPDRS items can be integrated in the same device, providing a more thorough surveillance of the patient's conditions (the *electronic diary*).

## V. Supplementary Materials

### A. Considerations on MDS-UPDRS Scoring

The MDS-UPDRS [5], [6] is universally employed to assess the course of PD after diagnosis. The evaluation encompasses six parts. Part I refers to mental state, behavior, mood, pain and autonomic dysfunctions. Part II is a patient self-assessment of several daily activities. Part IV addresses possible complications such as dyskinesias, whereas Part V and VI score the severity of the disease (Hoehn and Yahr scale^1^^1^The Hoehn and Yahr scale is a commonly used method to evaluate the disease progression and disability degree. It includes 5 integer plus 2 intermediate stages, with 5 being the worst case.). Part III, which is the most relevant for this work, is the clinical evaluation of several motor skills: speech ability, facial expressiveness, tremor, rigidity, sensitivity of the fingers, hand mobility, leg agility, ability to get up from a chair, posture and postural stability, gait characteristics, bradykinesia and hypokinesia. The clinician assigns an integer score between 0 and 4 according to the severity of the considered sign. This approach is not free of criticism, as it is know to lack repeatability, being affected by intra- and inter-rater variability [14]. Moreover, it only encompasses integer values, whereas a finer discretization could catch subtle variations (MDS recommendations, [26]). One of the key elements of this work is the definition of a proper ground truth for the classification task. According to the MDS guidelines, LA should be evaluated focusing the attention on speed, amplitude, slowing, hesitations and interruptions. However, a large margin of subjectivity does exist, leading to the already mentioned inter-rater variability issue: the same patient, examined by different clinicians, is likely to be scored differently [14]. The MDS recommendations suggest that the patient should be scored by at least three independent clinicians, even though this approach is seldom feasible in practical conditions. In our experiments, we obtained the clinical scores of each patient from four different neurologists (either directly or by inspection of video recordings), in order to provide a reliable data labelling to train and test the ML models. Another main novelty of this paper is that we have implemented an Artificial Neural Network (ANN) approach to face the discretization of LA, achieving a continuous index to be compared with the MDS-UPDRS score, averaged with respect to the four independent raters.

### B. Smartphone Sensors Evaluation

In our experiments, a Samsung S5 mini smartphone was employed. The characteristics of the embedded inertial sensors have been evaluated in order to assess their suitability for the specific data acquisition tasks. Whereas the dynamic range required for inertial sensors to match several human activities is well defined, no similar data can be found in literature related to LA or similar tasks. Hence, we have carried out a preliminary check, involving ten voluntary young male adults (age: $26.1 \pm 3.2$, body mass index: $22.8 \pm$ 2.1). This is a conservative case, as we expect elderly persons to perform the task with a lower movement intensity. They have been equipped with a SensorTile™ module from STMicroeletronics™, mounted on each thigh [27]. This state-of-the-art IMU exhibits a settable full scale range up to $\pm$ 16 g and $\pm$ 2000 dps for accelerometer and gyroscope respectively, and a 16 bit resolution. After being instructed, participants performed the LA task twice. The acceleration and angular velocity peaks turned out to be 1.13 g $\pm$ 0.35 and 236 dps $\pm$ 31 respectively. The 3D-accelerometer and 3D-gyroscope included in the Samsung S5 mini smartphone were found to have a sample frequency of 200 Hz, range $\pm$ 2 g and $\pm$ 2000 dps, resolution 40 mg and 60 mdps, respectively. Thus, embedded inertial sensors meet the data acquisition requirements. It can be noticed that most mid-range modern smartphones satisfy these (quite lossy) requirements. In any case, the sensor characteristics can be easily assessed prior to recommend the use of a given smartphone model for data acquisition. For the sake of completeness, also sample frequency and resolution are reported. We have checked that reported values were not limited neither by the Operative System nor the application employed. To this end, we have checked that no samples are lost, and we have visually inspected the signals, ensuring that no saturation occurred.

### C. Related Work

In recent years, thanks to the progress in the communication and information technology and the availability of low-cost sensors, the estimation of PD motor signs has received a lot of attention. A thorough review can be found in [28].

Several papers address data mining and Artificial Intelligence (AI) techniques to recognize the severity of PD cardinal motor signs, using data derived from wearable sensors. In 2015, [14], [17] enrolled 34 and 24 subjects respectively. Three Inertial Measurement Units (IMUs) were mounted on patient's chest and on each thigh in order to assess the LA task in clinical environment. Time- and frequency-domain features were extracted and selected in order to feed classification algorithms, i.e. Support Vector Machine (SVM) and k-Nearest Neighbors (kNN). Accuracy resulted in 43% in both studies; moreover, in [29] a correlation coefficient $r = 0.49$ is reported between the automatic scoring system and clinical MDS-UPDRS evaluation. Bradykinesia was evaluated in [21] through a specific smartphone application tested on 14 people with PD. Different MDS-UPDRS part-III items were addressed, i.e. 3.4, 3.6 and 3.8. Focusing on the LA task, the best correlation with the clinical MDS-UPDRS score was found with leg movement power ($r=-0.5$, $p=0.015$). No classification was performed. Recently, in [22] a six-month clinical trial is reported, conducted on 44 people with PD to assess many MDS-UPDRS motor tasks by means of a smartphone in home environment. Despite LA was not included into the study, the gait task may be partially considered an indicator of bradykinesia; authors found an Intra-class Correlation Coefficient ($ICC$) of 0.88 with gait score. Moreover, in [18] 19 PD subjects were monitored with ankle-mounted inertial sensors for LA evaluation and treatment response. Time- and frequency-domain features were computed to feed different classifiers, i.e. SVM, Decision Tree (DT), linear regression model. Performance were expressed in terms of $ICC$, correlation coefficient and Root Mean Square Error ($RMSE$) with respect to the MDS-UPDRS bradykinesia score (item 3.14); $ICC=0.89$, $r=0.83$ and $RMSE=0.53$ were reported. At last, in [23] 50 people with PD were tested while wearing IMUs on each ankle for LA quantification. A fuzzy logic inference model was built exploiting both the most meaningful features and rules based on MDS-UPDRS item 3.8 recommendations. Unfortunately, classification results in terms of accuracy, $r$, $ICC$ and $RMSE$ are not reported. It is worth noticing that all the aforementioned studies present some limitations: reduced sample dimension (maximum 44 people with PD), dedicated hardware (only one study employs smartphones), number of sensors (most studies use three sensors). Furthermore, some relevant dataset details (e.g. cardinality of MDS-UPDRS classes) are not reported.

### D. Inter-Rater Variability

**Fig. 6.**
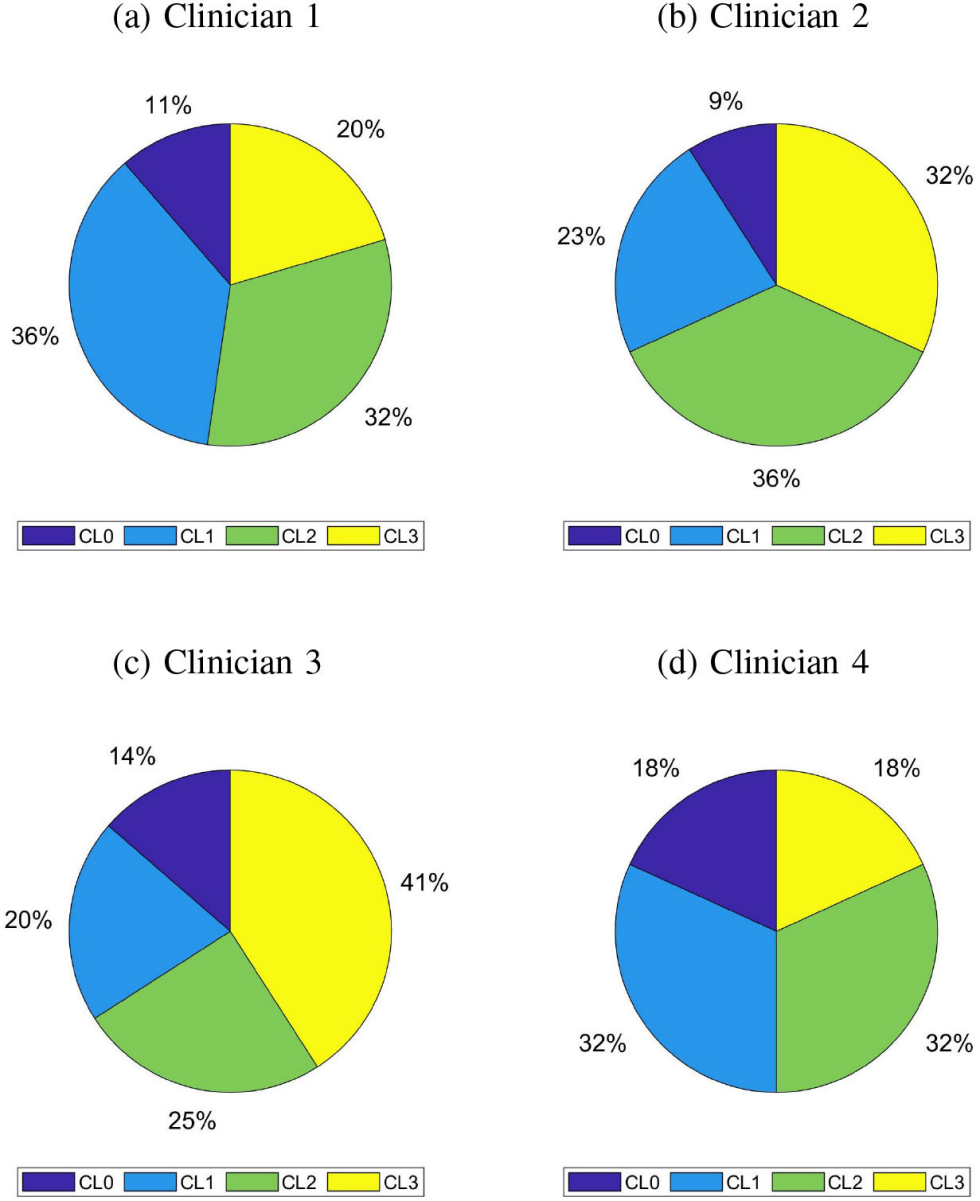
For each evaluating clinician, score distribution among UPDRS-part III Leg-Agility score (CLx stands for score x, x = LA score)

The possible disagreement in assigning MDS-UPDRS scores can be justified by the complexity in discriminating between adjacent classes, given that the clinicians are required to pay attention to several different aspects. Hence, it is worth questioning whether differences between clinical and automatic scores is due to non-proper feature selection or is intrinsic in the data. This dilemma is also addressed in [24], where the authors conjectured that automated methods may turn out to be more reliable than clinicians themselves for this reason. To this end we have investigated the neurologists agreement per MDS-UPDRS class. The pie charts reported in Fig. 6 show the distribution of scores for each clinician. This suggests that the discordance between the clinical score and that provided by our algorithm may be mainly due to this inter-rater variability. As discussed in Section III-A, the correlation between the ANN continuous score and the average clinical score provided by four neurologists by examining each patient either directly or via the videotaped leg movement is higher than the best clinicians agreement. Given that automated score performed better than each single clinician, we suggest that a continuous score given by a ML-based tool could be used as a tool to overcome intra- and inter-rater variability.

#### 1) The Patient's and Caregiver's Points of View

Achieving a fine monitoring of the disease progression, without requiring the patient to face stressing, costly and impractical movements from home, represents the obvious main advantage for patients. A fine drug posology adjustment can extend the years of good disease control, yielding an improved quality of life. The patient feels safer and under control, and this promotes more stable mood and more residual autonomy in a pathology, which exhibits a large incidence of depression and anxiety. As for acceptance, have interviewed about 100 patients, and the large majority of them agrees that a noninvasive, easy-to-use, unobtrusive, low-cost technology would be greatly appreciated. Using the smartphone as a data collector exhibits pros and cons from the patient's point of view. It is a relatively high weight device, but on the other hand, it is widespread and does not imply significant additional cost. This task has demonstrated to be feasible and little bothersome.

#### 2) The Professional Point of View

Neurologists are well aware that yearly, pre-scheduled visits do not enable an adequate patient's follow-up, especially in intermediate stages of the disease, and are willing to adopt technological support. On the other hand, the relevant information should be condensed in very concise periodic reports. It must be noticed that, at present we lack a standardized protocol and infrastructure able to transmit and store this information [24]. Hence, the design of scalable, web-based architectures, managing heterogeneous types of information and standards, compliant with regulatory and security rules for medical data, is a crucial issue. The cost effectiveness of such an infrastructure, in terms of improved health and reduction of hospital admissions, needs also to be quantified.
